# Constructivism: reflections on twenty five years teaching the constructivist approach in medical education

**DOI:** 10.5116/ijme.5763.de11

**Published:** 2016-06-25

**Authors:** Reg Dennick

**Affiliations:** 1 Medical Education Unit, School of Medicine, University of Nottingham, UK

**Keywords:** Constructivism, reflections on teaching, constructivist approach, medical education

## Introduction

There is no single overarching theoretical framework that accounts for how we learn in all situations. There are many theories of how we learn from our experiences and there are many theories of what experiences are.  We know that we do learn and that we have knowledge but there is no consensus on the relationship between the mechanism by which our brains learn and the optimal way in which we should be taught. In other words there is no necessary connection between epistemology and pedagogy. We seem capable of learning from a wide variety of pedagogical processes and although there is empirical evidence that some ways of learning are more effective than others in specific situations no one pedagogical method dominates. However, reflecting on twenty five years as a medical educationalist I have come to the conclusion that there is one framework that makes more connections between different epistemological and pedagogical theories than others and that could have support from neuroscience. I wish to explore the connections between a variety of educational, communication and psychotherapeutic processes and try to show that constructivism holds the promise of providing some unity to the practice of education and learning.

### The constructivist model

The constructivist theory of learning, whose philosophical origins are frequently ascribed to Kant and whose educational origins to Piaget, is based on the premise that the act of learning is based on a process which connects new knowledge to pre-existing knowledge. I do not intend to go into all the arguments surrounding this theory and to describe the variety of constructivist models that have been created. The reader is referred to the extensive literature on the subject.^1^^-^^6^    For the purposes of my argument it is simply necessary to acknowledge some basic assumptions underpinning the theory from which important connections to other educational theories can be made as well as to therapeutic practices, neuroscience and even the nature of scientific knowledge.

Possibly the most well-known articulation of the underlying assumption of constructivism is the famous quotation of Ausubel: “The most important factor influencing learning is what the learner already knows”.^7^ In many years of teaching teachers how to teach I have  found many people have an intuitive grasp of this fact and tailor their teaching to take account of the background knowledge of their students. Piaget expressed it differently by stressing that experience is constantly being ‘assimilated’ or filtered through pre-existing concepts.^8^    New knowledge is therefore interpreted by existing knowledge and then connected to existing knowledge. The implications of this conception are manifold and spread beyond conventional education into interpersonal communication and psychotherapy. In addition this phenomenon begs the question of how the process is conducted and manifested in the brains of individuals.

The statement of Ausubel^7^ automatically leads to a pedagogical intervention even if it is merely ascertaining the prior knowledge of a learner by asking questions. Piaget avoided suggesting detailed pedagogy but certain ways of teaching flow from his assumptions. He stressed that learning was fundamentally about interacting with the world in order to explore the ‘rules of the game’ and to discover the causal relationships between events.  This leads to an active pedagogy involving exploration, experience and experimentation. Dewey aptly summarized this idea by saying that learners should be *actors rather than spectators*.^9^ Others have extended this concept to suggest that individals  interact with the world in order to extract meaning from it and to construct a coherent and consistent cognitive model. Another way of saying this is that our brains are programmed to support our survival. From an evolutionary perspective organisms have evolved brains which create and internalize an increasingly complex and accurate model of the world. Based on our experience of interacting with the world we have each created our own unique model of reality. Nevertheless we are social beings and we cannot ignore the power of social interactivity that has influenced this process, a concept emphasised by the Russian psychologist Vygotsky.^10^ Other human beings influence the way in which we construct our models giving rise to the teacher and to the various social processes of pedagogy.

Model building in the individual,  based on interactivity with the world, is the result of a cognitive process which involves the experience of the world being assimilated and filtered through prior knowledge as previously described. If sense or meaning can be attached to the experience then the experience fits with existing cognitive structures (Piaget’s ‘schemas’).  However, if the experience does not make sense then a feeling of dissatisfaction or cognitive dissonance can ensue in which the individual will seek to reach some sort of mental equilibrium by exploration or questioning. At this point the individual may make use of the faculty of imagination to suggest or hypothesise why the experience is problematic. It is the existence of human imagination, the ability of each of us to interrogate our mental model, to speculate and to ask questions that is one of the  hallmarks of our intelligence and our capacity to survive. Albert Einstein famously stated that ‘imagination is more important than knowledge’ since he realized that ultimately all knowledge is derived from an initial process of imagination that is subsequently tested against the world.  As individuals we can simulate reality by asking ourselves ‘what if?’ questions.  We can perform acts in our imagination before doing them in reality and risking having our genes deleted from the gene pool.^11^ Our imagined conjectures can be tested by seeking other experiences and by so doing we can resolve the dissonance and elaborate our learning and our mental model.

### Constructivism and scientific reasoning

You may notice that what I have just described,  as the individual attempts to construct a more meaningful and coherent mental model,  is what is often called hypothetico-deductive reasoning. When confronted with experience hypotheses are created via inductive reasoning and human imagination. These hypotheses are then tested by exploration, further experience or deliberate manipulation of the world coupled to and  processed by deductive reasoning. Hypotheses are then either rejected or supported,  leading to the elaboration of knowledge.  Hopefully the reader should now see that what the constructivist model of learning describes in the individual is what is commonly known as the ‘scientific method’.^12^ Driver and many other constructivists have frequently asserted that the individual learner behaves like a scientist in seeking to make sense of the world.^13^Indeed Glopnik has gone so far as to assert that even the baby in the cradle is a little scientist testing out hypotheses about reality.^14^

In summary we assert that the constructivist model is built on the premise that the brain naturally attempts to extract meaning from the world by interpreting experience through existing knowledge and then building and elaborating new knowledge in a process identical to hypothetico-deductive reasoning or the scientific method. 

### Clinical diagnostic reasoning

Diagnostic reasoning is one of the key cognitive skills that doctors need to acquire and medical schools need to teach. It has many characteristics in common with scientific reasoning and hence is connected to the constructivist model. When a patient presents with a problem the doctor will  begin a process of hypothesis formation aided by information derived from the patient’s history and examination findings. Pattern recognition and ‘gut feelings’ are important here but very much depend on the range and quality of the mental models of illness presentation that they have built up. This initial phase  can be seen as an inductive process in which sensory information is assimilated through the pre-existing knowledge of the clinician. The hypotheses, or differential diagnoses, next require testing to see which ones can be supported,  falsified and  eliminated by the acquisition of further evidence. This might involve more history taking and examination or the ordering of investigations such as blood tests or radiographs. The results obtained can be used to eliminate some hypotheses and can potentially lead to a final diagnosis of the problem.  This phase of the process is characterized by deductive reasoning and the whole diagnostic process as hypothetico- deductive reasoning,  which can hopefully be seen as identical to the scientific method as previously described.

Of course many things can go wrong in this process.  Lack of background knowledge and experience will inhibit the assimilative inductive phase and reduce hypothesis formation from the imagination. Bias can influence the hypothesis testing phase by only  looking for evidence to support a diagnosis rather than attempting to falsify one. Interpreting the results of investigations can also be  influenced by a lack of background knowledge and experience. There are a myriad factors that affect the outcome of the diagnostic process and there are many ways of teaching this skill. Nevertheless it is important from a medical education perspective that students are made aware of the constructivist nature of the hypothetico-deductive process as the underlying engine of diagnostic reasoning.

Now that the constructivist framework has been described and its relationship to scientific method  has been outlined it is useful to look at other theoretical models to see if there are any connections that can be discerned.

### Students’ theories

It is a remarkable and well evidenced observation that students learning science have their own mental constructs or ‘theories’ and will articulate them when asked to explain the phenomena they are exposed to.^15^ This can be explained by the constructivist model in that experiences will be filtered through limited prior knowledge followed by imaginative attempts to provide a plausible explanation. The student comes to school with years of personal experience of heat, light, gravity, forces, motion, solids, liquids, gases, energy, electricity, plants,  animals and people.  It is not surprising that strongly held theories about the nature of the physical, biological and social world have been elaborated by the student even before any scientific teaching is encountered.

Since the student is at the heart of learning good pedagogy suggests that it is essential that teachers make the effort to try to understand the student’s point of view. Teachers should create learning situations that encourage students to bring out their ideas. It is here that the ‘social constructivist’ approaches described by Vygotsky become important. The individual develops conceptual understanding via the social sharing of meanings and intellectual debate. Thus as far as possible scientific and medical learning should be a group activity with opportunities to discuss ideas, make hypotheses and devise ways of testing them.  The teacher should facilitate the developing conceptual understanding of students by providing examples of cognitive conflict as well as emphasising how well the ‘scientific’ concepts explain phenomena in comparison to the more contradictory  theories of the students.  Of course the aim of teaching is to replace the ‘erroneous’ beliefs of the student with the ‘correct’ evidence-based scientific ones. Problem based learning is an ideal way to create this teaching environment.^16^

However, children become adults and although they may then be considered  mature thinkers , (‘logical operators’ to use Piaget’s terminology), nevertheless it is clear from many studies that they still have scientific misconceptions.^17^ Medical students can be considered to have broken through the immature phase of science education and clearly they have successfully learned the correct scientific theories and concepts required to get into medical school.  Nevertheless, that doesn’t mean they are immune from still having personal theories and erroneous beliefs that might get in the way of acquiring the received evidence-based wisdom provided by their teachers.

### Lay theories

Adults,  with little knowledge of science and medicine beyond an elementary education,  will have ‘lay beliefs’ concerning the anatomy and physiology of the body and of the causation of illness,  and medical students and practitioners will interact with patients holding such beliefs.^18^^-^^21^

Lay beliefs are therefore a component of the mental models that individuals have made and hence are part of the constructivist framework. Health care professionals need to be aware of lay theories when engaging with patients as they can have an important impact on health outcomes.

However, when we move into the area of lay beliefs we are entering sociological territory and the metaphor of construction is used in a radically different way. In Berger and Luckman’s^22^ work ‘The Social Construction of Reality’ it is implied that reality itself is very much a social construction and that there is no single, coherent, real world. This ‘Postmodern’ viewpoint asserts that individuals experience reality as multifaceted and contradictory and rejects scientific ‘objectivity’. This extreme view of constructivism can lead to a cognitive relativism that is a long way from the  scientific approach.

Illness occurs within a culture that fundamentally shapes how that illness is experienced. Anthropologists have provided a rich source of examples of the way in which lay beliefs about illness are part of the social fabric of all societies.  Many studies in contemporary society demonstrate the continuing existence of a wide range of health beliefs that are seen as causative factors in illness.^23^  It is tempting to suggest that these constellations of causal factors constitute ‘theoretical frameworks’ but they do not exhibit a high degree of consistency, order, stability and rationality and respondents often maintain and use contradictory models without recognising a logical inconsistency between them.

It may appear paradoxical that one of the claims of constructivism is that the brain uses a process of ‘scientific’ enquiry to try to make sense of the world but that individuals can then come up with and sustain ‘non-scientific’ beliefs or theories. Although the process may follow a scientific logic the final outcomes can clearly be totally unscientific and result from a lack of background knowledge, personal bias and an uncritical interpretation of evidence. Thomas Kuhn famously proposed that even scientists can be resistant to evidence that could lead to theory change and it sometimes takes a revolution to enable ‘paradigm shifts’ to take place.^24^  Individuals can be equally resistant to evidence that might change their belief systems and might need their own personal revolution.

Nevertheless the real and potential gulf between lay beliefs and medical knowledge is a major factor in the therapeutic effectiveness of the doctor-patient relationship.  Doctors need to understand the constructions of their patients and need to speak to them using language they can understand.^25^ Eliciting the patient’s ideas is an essential step in this process although this can be another source of confusion since what a lay person understands by a particular medical term maybe entirely different from the doctor’s understanding.^26^

In the medical field interpersonal communication or ‘communication skills’ is very important. The ability to listen to an individual, to ask them questions, to interpret what they are saying and to give them information and advice in a way that they can understand is a major feature of good medical practice which in one sense is educational. At the heart of it is an engagement with an individual  that is analogous to the learner centred approach and hence is described as patient centred. As in constructivist pedagogy it involves finding out the background knowledge of the patient and then communicating with them and educating them in a way that they can understand. If constructivist pedagogy is used to establish prior knowledge it is also used in helping the patient to construct their understanding of their condition and to help the doctor to establish potentially new behaviours in the patient which will be of medical benefit. In many ways this is fundamentally associated with a teaching process and the Cambridge-Calgary method is a well established formulation of this method.^27^

### Experiential learning

The experiential learning theory developed by David Kolb^28^ cites Piaget as a precursor in addition to acknowledging the influence of the social factors on learning identified by Vygotsky. Therefore it comes with good constructivist credentials.  However, Kolb’s framework has been interpreted and misinterpreted by so many individuals over the years that it is sometimes difficult to see where constructivist concepts can be found within it. Because experiential or ‘non-formal’ learning frequently happens in a haphazard and unstructured way,  with individuals having raw experiences in working environments,  it is not always obvious that there is any pedagogy involved. But those ‘concrete experiences’ still have to be assimilated through pre-existing cognitive constructs.  It is here that the social constructivist  role of others becomes important,  either as mentors who deliberately foster ‘reflection’ or merely fellow learners who can discuss and refine understanding.  Kolb’s ‘abstract conceptualisations’ become the mental models discussed earlier and ‘active experimentation’ becomes the deductive reasoning process associated with questioning and active learning.

### Humanistic theories

In the field of education humanistic theories of learning have contributed ideas and practices that complement constructivist and behaviourist models of learning.  In particular the humanistic theories of Rogers and Maslow emphasise the importance of acknowledging the individual and starting from their standpoint in either a therapeutic or educational process.^29^^,^^30^ This leads to human-centred or learner-centred approaches to education where the needs of the learner become the heart of the educational process. In addition one of the principles of adult learning, sometimes termed ‘andragogy’, is that the life experiences  and background knowledge of adult learners becomes an important educational resource.^31^ With adult learners, therefore, we build on and respect their prior knowledge,  which may have been acquired from their own personal needs. But as we have previously established starting from where the learner is and building on their knowledge is a fundamental tenet of the constructivist model. Thus we can establish a strong connection between constructivism and learner-centred approaches.

### Personal construct theory and cognitive behavioural therapy

George Kelly’s Personal Construct Theory^32^ is built on the premise that ‘all men are scientists’ and have constructed specific mental constructs and beliefs based on their experiences that make them what they are.  It also posits that mistaken constructs can be created which can cause mental problems and inappropriate behaviour. Constructivist psychotherapy and Cognitive Behavioural Therapy are similarly based on the premise that individuals may have constructed inappropriate behaviours and beliefs.^33^ Therapy involves challenging these ‘cognitive distortions’ to normalize behaviour.

### Neuroscience

Social constructivists often stress that as social beings our knowledge is disseminated amongst other human beings and also stored within physical locations such as libraries and the internet.  But we cannot lose sight of the fact that learning and memory are ultimately processes that take place in individual human brains. Therefore what does the constructivist metaphor mean in terms of neural structure and processing? Is the construction of knowledge paralleled by the construction of neural structures and do the pedagogical processes suggested by the epistemology of constructivism enhance these constructions?

There is considerable evidence that our perceptions of reality and our own mental states are,  in fact,  constructions.  Images projected onto the retina are analysed and processed by a variety of mechanisms in the brain and what we ‘think’ we see is largely a creation of mental processing. The central image on the fovea,  scanned by unconsciously experienced saccadic eye movements, constitutes the centre of a perception whose peripheral elements are entirely ‘filled in’ by the brain. We do not therefore perceive reality as it actually is but we perceive a construct based on  a probabilistic model of reality created by the brain. Our brains are constantly predicting what is out there in the world and by a series of ‘top down’ processes they fill in the sensory data we are receiving to create what Clark has called a ‘controlled hallucination’.^34^

Further illustrations of how the brain constructs our perceptions, thoughts and even feelings have been provided by the work of Ramachandran.^35^^,^^36^  Oliver Sacks has also written eloquently about how individuals who have tragically suffered from damage to specific regions of the brain can reconstruct their cognitions,  often in strange and unusual ways.^37^ The key conception that derives from these studies and observations  is that the brain actively ‘fills in’ gaps in perception, sensation and cognition with constructs that attempt to maintain some sort of mental cohesion, even if the results can sometimes be anomalous.

It has been argued that constructivism is underpinned by mainstream theories of cognitive neuroscience: it is how our brains work when we are learning. According to  Quartz and Sejnowski^38^ the cerebral cortex has evolved to maximize its structure and function through constructive learning. In addition the importance of active learning methods,  as recommended by constructivist pedagogy,  is supported by studies of   neurogenesis in the adult brain.  Neurogenesis continually occurs in the dentate gyrus area of the hippocampus of the human brain, a region well known for its part in learning and memory.^39^ Furthermore it is now established from mouse models that activity in an enriched living environment stimulates neurogenesis and results in increased synaptic connectivity.^40^ The implication of these and other studies suggests that learning is a physically constructive process in the brain which is enhanced by active learning.

## Conclusions

The key principle of constructivism in education is that learning is always a building process whereby  new knowledge can only be added on to and understood in terms of existing knowledge. The ramifications of this concept are significant. Constructivist epistemology suggests constructivist pedagogy such as always checking and activating prior learning. Constructivism implies that hypothetico-deductive reasoning is a process we all engage in when trying to understand the world. The scientific method and diagnostic reasoning  are essentially constructivist. Constructivism underpins many human interactions where dealing with and recognising the prior knowledge and personal constructs of an individual are important such as teaching in general, communication skills in medicine and some types of psychotherapy. Finally it is increasingly being suggested that the physical structure of the brain and its processes provides a neuroscientific rationale for constructivist cognition implying that certain pedagogical methods such as active learning should be encouraged. All medical and health science educators should be aware of the fundamental principles of constructivism and the extent of its influence on educational theory and clinical practice.

### Conflicts of Interest

The author declares that he has no conflict of interest.
